# Vitiligo Skin Biomarkers Associated With Favorable Therapeutic Response

**DOI:** 10.3389/fimmu.2021.613031

**Published:** 2021-03-05

**Authors:** Qianli Yang, Guohong Zhang, Mingwan Su, Gigi Leung, Harvey Lui, Pingyu Zhou, Yan Wu, Joshua Zhou, Jinhua Xu, Xuejun Zhang, Youwen Zhou

**Affiliations:** ^1^Department of Dermatology and Skin Science, University of British Columbia, Vancouver, BC, Canada; ^2^Department of Dermatology, Huashan Hospital, Fudan University, Shanghai, China; ^3^Department of Pathology, Shantou University Medical College, Shantou, China; ^4^Shanghai Skin Hospital, Tongji University, Shanghai, China; ^5^Department of Dermatology, First Hospital, China Medical University, Shenyang, China; ^6^Faculty of Dentistry, University of British Columbia, Vancouver, BC, Canada; ^7^Institute of Dermatology, Anhui Medical University, Hefei, China

**Keywords:** vitiligo, RNA sequencing, biomarkers, response to therapy, phototherapy, tacrolimus, therapeutic markers

## Abstract

Vitiligo is an acquired depigmentation skin disease caused by immune-mediated death of melanocytes. The most common treatment for vitiligo is narrow band ultraviolet B phototherapy, which often is combined with topical therapies such as tacrolimus. However, patients’ responses to these treatments show large variations. To date, the mechanism for this heterogeneity is unknown, and there are no molecular indicators that can predict an individual patient’s response to therapy. The goal of this study is to identify clinical parameters and gene expression biomarkers associated with vitiligo response to therapy. Six patients with segmental vitiligo and 30 patients with non-segmental vitiligo underwent transcriptome sequencing of lesional and nonlesional skin at baseline before receiving combined UBUVB and tacrolimus therapy for 6 month, and were separated into good response and bad response groups based on target lesion achieving > 10% repigmentation or not. Our study revealed that treatment-responsive vitiligo lesions had significantly shorter disease duration compared with non-responsive vitiligo lesions (2.5 years vs 11.5 years, p=0.046, t-Test), while showing no significant differences in the age, gender, ethnicity, vitiligo subtype, or disease severity. Transcriptomic analyses identified a panel of 68 genes separating the good response from bad response lesions including upregulation of immune active genes, such as CXCL10, FCRL3, and TCR, Further, compared with vitiligo lesions with long disease duration, the lesions with short duration also have much higher level of expression of immune-active genes, including some (such as FCRL3 and TCR genes) that are associated with favorable therapeutic response. In conclusion, our study has identified clinical parameters such as short disease duration and a panel of immune active and other gene expression biomarkers that are associated with favorable response to immune suppressive NBUVB + tacrolimus therapy. These markers may be useful clinically for individualized therapeutic management of vitiligo patients in the future.

## Introduction

Vitiligo is an acquired depigmentation skin disorder that affects 0.5%–2% of world’s population (1). Patients with vitiligo develop white patches on their skin, including in visible areas such as the face, neck, hands, and forearms. As a result, the quality of life of vitiligo patients can be severely reduced (2–5). Clinically two major types of vitiligo are recognized: segmental vitiligo (SV), which affects a localized asymmetrical area of the body, and non-segmental vitiligo (NSV), the more common subtype, which often involves multiple body sites in a symmetrical fashion.

In addition to loss of melanocytes, vitiligo patients have increased risk of developing other autoimmune diseases, such as diabetes, thyroiditis, rheumatoid arthritis, alopecia areata, lupus erythematosus, and adrenal insufficiency (6, 7). The pathogenesis of vitiligo is unknown, although previous studies identified multiple factors that are potentially involved in the development of vitiligo, including genetic predisposition (such as polymorphisms in genes involved in the immune response and in melanogenesis) (8–14), inducible heat shock protein 70 (15, 16), activation in vitiligo lesions of adaptive immunity [such as CXCL10 (17)] and innate immunity [such as NK cells (18)], environmental factors (such as exposure to certain chemicals (19–21), abnormalities in metabolic and oxidative stress responses (22–24), abnormalities in melanocyte cell adhesion (25), and neurogenic inflammation (9, 10, 26–28) It is unknown if segmental and non-segmental vitiligo involve the same pathogenic mechanisms.

Treatment of vitiligo is challenging. Repigmentation is often incomplete, and requires prolonged therapeutic exposure and careful optimization of treatment protocols (29–31). At present, the most widely available therapy is narrow band ultraviolet B phototherapy (NBUVB) (30, 31), and topical calcineurin inhibitors such as tacrolimus (32, 33), often used in combination (34–41). These therapies are limited in that they are modestly effective (31), require prolonged maintenance, and can be associated with side effects, such as accelerated photo-aging, and the development of photo-lichenoid papules (42). Numerous novel therapies are under investigation, mostly by targeting the immune response regulators, such as JAK inhibitors (43–46), are under investigation.

At present, there are no reliable methods for predicting a given individual patient’s treatment outcomes to vitiligo therapies, apart from the well-established clinical observations that facial vitiligo and pediatric patients are more responsive to therapy. Adult vitiligo patients show a high degree of heterogeneity in response to therapy that is not explained by these factors. To date there are no biomarkers shown to predict treatment response to vitiligo therapy.

In the current study, we performed a clinical-transcriptomic correlational analysis to systemically examine the molecular landscape and specific biological pathways of vitiligo skin microenvironment prior to the initiation of NBUVB phototherapy, and evaluate if any clinical features, such as disease subtype and lesional duration, or gene expression features are correlated with a favorable response to combined photo-topical therapy.

Our results revealed that adult vitiligo lesions with recent onset and the vitiligo lesions with biomarkers of active innate and adaptive immune responses respond more favorably to the immune suppressive combined photo-topical therapy. Therefore, vitiligo therapy should be started as early as possible to suppress active innate and adaptive immune activities.

## Materials and Methods

### Vitiligo Patient Clinical Information and Skin Biopsies

With approval from the Clinical Ethics Board of University of British Columbia, 36 vitiligo patients (30 non-segmental vitiligo and 6 segmental vitiligo) attending the outpatient dermatology clinics at the Skin Care Center of Vancouver General Hospital and nine healthy volunteers participated in this study.

The baseline clinical information was collected from each individual, including demographics, general medical, and medication history, vitiligo specific information such as onset, duration, extent of skin involvement, anatomical distribution, previous therapies and associated responses. A target lesion at least 2 cm in diameter was selected from a non-cosmetically sensitive area of the skin such as the torso or the proximal extremities and photographed. With informed consent, two 4 mm punch full-thickness biopsies were performed, one from the lesional skin 1 cm inside border of the target lesion (lesional skin, or LS), the other, nonlesional skin 1 cm outside of the lesional border (nonlesional skin, or NLS). The biopsies were bisected, with one portion placed immediately in RNA Later solution (Life Labs) and stored at -20° until further use. The other portion was placed in formalin for histological assessment. One subject’s lesional skin biopsy did not contain significant reduction of melanocytes, thus the diagnosis of vitiligo was not confirmed, and was excluded from the rest of the analyses. For the nine healthy volunteers, a single 4 mm punch biopsy was performed from the torso or the proximal extremities.

After the biopsies were obtained, the vitiligo patients received NBUVB-tacrolimus combination therapy that included NBUVB phototherapy three times per week according to the protocol previous reported (31, 42) and topical application of tacrolimus 0.1% ointment twice daily on non-phototherapy days. The target lesions were assessed every three months for the percentage of re-pigmentation in comparison to the baseline photographs until the end of 6th month. Ten percent (10%) re-pigmentation was used as the criteria separating the responsive from non-responsive groups. This was chosen to maximizing the statistical power of the study as it roughly separated the vitiligo patients undergoing treatment to two groups with similar sizes.

### RNA Extraction and Transcriptome Sequencing

Bulk RNA was extracted from each skin biopsy using the RNeasy^®^ Fibrous Tissue Mini Kit as we had described previously (18), and used for transcription sequencing using the Novo Gene Ilumina platform (HiSeq PE150, Tianjin China), generating at least 30 million clean reads for each sample. The abundance of each transcript is normalized to the total number of transcripts and the length of the transcripts and expressed in FPKM. The averages of the depigmented biopsies (LS), nonlesional biopsies (NLS), and control healthy normal skin (HNS) were calculated for all of the coding genes of the human genome. All of the genes showing more than 2 fold differences (up or down regulated) between the LS and NHS (p<0.05 two tailed t test) were identified (723 genes in total) (See **Supplemental Table 1**). This table also shows the details of expression of these genes between NLS and HNS, and between LS and NLS (ratios and p values).

The R, IPA, Reactome PA, and SPSS packages were used for datamining and statistical analyses.

## Result

### Vitiligo Patient Characteristics and Therapeutic Response

The demographics and vitiligo-specific parameters are summarized in **Table 1**. There was a slight female dominance (1.12 F: M ratio), with an average age of 44.5 years (range 14.2 to 72 years) at time of assessment, and 37.2 years at time of vitiligo onset (range 14 to 71 years). The average vitiligo severity is 8.4% BSA (body surface area, range 1% to 30%). Thirty patient had non-segmental vitiligo whereas six patients had segmental vitiligo. There were 14 Caucasians and 22 Asians (including 11 South Asians and 11 East Asians).

**Table 1:** Clinical Information of vitiligo patients.

ID #	Sub-type	Sex	Ethnic Origin	Age at Onset (yr)	Age at Biopsy (yr)	Vitiligo Duration (yr)	Vitiligo Severity (BSA %)	Location of Biopsied Lesion	Responsive to therapy?
Vit 01	NSV	F	SA	30	31.5	1.5	5	Arm	~
Vit 02	NSV	F	SA	30	31.5	1.5	5	Arm	~
Vit 05	NSV	F	SA	26	30	4	2	Arm	no
Vit 12	NSV	M	Cau	70	72	2	2	Hand	no
Vit 13	NSV	M	SA	54	54.5	0.5	5	Abdo	yes
Vit 14	NSV	M	Chi	10	17	7	11	Leg	no
Vit 20	NSV	F	SA	35	39	4	10	Abdo	no
Vit 21	NSV	M	Chi	28	38	10	30	Arm	no
Vit 22	NSV	M	Chi	19	69	50	20	Back	no
Vit 27	NSV	M	Cau	35	55	20	10	Torso	~
Vit 28	NSV	M	Cau	43	43.75	0.75	5	Torso	~
Vit 29	NSV	F	Cau	14	14.2	0.2	10	Arm	yes
Vit 31	SV	F	SA	57	58	1	3	Neck	~
Vit 33	NSV	F	Cau	23	33	10	10	Knee	no
Vit 34	NSV	M	SA	8	8.5	0.5	22	Abdo	yes
Vit 35	NSV	M	SA	30	70	40	10	Back	no
Vit 36	SV	M	Chi	51	51.6	0.6	2	Neck	yes
Vit 37	NSV	F	SA	55	56	1	15	Torso	no
Vit 39	NSV	M	Cau	64	65	1	7	Neck	yes
Vit 40	NSV	F	Chi	71	71.4	0.4	2	Abdo	yes
Vit 42	NSV	M	Chi	36	59	23	30	Hand	yes
Vit 43	SV	F	Cau	36	36.4	0.4	2.5	Face	yes
Vit 44	SV	F	SA	47	48	1	3	Neck	no
Vit 45	SV	M	Chi	26	26.5	0.5	5	Buttock	yes
Vit 47	NSV	F	Cau	17	32	15	5	Axilla	~
Vit 48	NSV	M	Cau	12	27	15	10	Back	~
Vit 49	NSV	F	Cau	63	75	12	3	Arm	no
Vit 50	NSV	F	Cau	58	59	1	6	Back	~
Vit 51	NSV	F	Chi	36	46	10	5	Back	no
Vit 54	NSV	M	Cau	43	53	10	5	Back	no
Vit 55	NSV	F	Kor	28	28.5	0.5	20	Flank	yes
Vit 56	NSV	F	SA	40	40.2	0.2	6	Shoulder	yes
Vit 57	NSV	M	Cau	50	60	10	8	Arm	~
Vit 58	SV	F	Cau	63	63.3	0.3	3	Neck	~
Vit 59	NSV	M	Chi	15	25	10	3	Torso	no
Vit 61	SV	F	Chi	15	15.75	0.75	1	Neck	~

SV, segmental vitiligo; NSV, nonsegmental vitiligo; Cau, Caucasians; Chi, Chinese, Kor, Korean; SA, South Asian; BSA, Body surface area; ~ not available.

Of the 36 vitiligo patients, 11 patients did not undergo treatment, and were not included in the therapeutic response analysis. The remaining 25 patients underwent NBUVB-tacrolimus combination therapy. The NBUVB phototherapy was administered according to protocols previously reported by Hemzavi et al. (31). On the days when not on NBUVB phototherapy, the patients applied topical tacrolimus 0.1% ointment BID. The repigmentation of the target lesions was assessed at three-month intervals, with 11 achieving more than 10% repigmentation at 6 months compared with photographs taken at the baseline.

The 11 responders and the 14 non-responders (achieving less than 10% repigmentation at 6 months) did not show any significant difference in sex (female 54.5% vs 53.3%), ethnicity (Caucasian/Asian ratio 0.38 vs 0.36), age at onset (38.9 yrs vs 35 yrs), age at biopsy (41.4 vs 46.4), vitiligo severity at baseline (10.1% BSA vs 8.6% BSA), vitiligo subtype composition (SV/NSG ratio 3/8 vs 1/13) (Chi-square test, p>0.05), or anatomical location of the target lesions (acral in 1/10 patients vs 1/13 patients)(Chi-square test, p>0.05). However, there was a significant difference in the lesional duration between these two groups. The responding lesions had an average duration of 2.5 years (range 0.2 to 23 years) whereas the average duration of non-responding target lesions was 8.6 years (range: 1 to 40 years) (two tailed t test, p=0.047) (**Table 2**).

**Table 2:** Correlational Analysis of Response to NBUVB-Tacrolimus Therapy.

Parameters	Favorable Response to Therapy	Non-Favorable Response to Therapy	Response Not assessed	p
Sex (% males)	54.5%	53.30%	36.4%	ns
Ethnicity (Caucasians vs Asians)	3 vs 8	4 vs 10	7 vs 4	ns
Age at biopsy	41.4	46.4	43.3	ns
Age at onset	38.9	35.0	37.3	ns
Duration of lesion	2.5	11.4	6.1	0.047
Vitiligo severity (%BSA)	10.1	8.6	5.5	ns
Segmental vs Non-segmental ratio	3 vs 8	1 vs 13	3 vs 8	ns
Acral vs non-acral	1 vs 10	1 vs 13	0 vs 11	ns

BSA, body surface area; ns, not significant.

### Transcriptomic Features of Lesional and Non-Lesional Vitiligo Compared with Healthy Normal Skin

Complete transcriptome analysis revealed 19,041 coding transcripts with detectable expression in at least one of the skin biopsies. Compared with normal healthy skin, there were 392 and 331 genes with increased and decreased expression in vitiligo lesional skin, respectively (>2 fold change, average level of each group, p<0.05, two tailed t test) (See **Supplemental Table 1** for details). The upregulated genes in vitiligo lesional skin include markers of inflammation (such as MARCO, NLRP10, PTGS2, and IL36G), oxidative response genes (DUOXA2), and NK cell receptors (NCR3LG1), revealing presence of activated immune response in vitiligo lesions.

The down regulated genes include those involved in melanin synthesis and melanocyte development (TYRP1, PMEL, DCT, TYR, MLANA, and MC5R), neural crest cell development (PLP1, SYN2) and lipid and surfactant metabolism (ACOT1, ACOT2, ACOT4, ACOX2, ACSBG1, ACSM3, ACSM6, PCSK2, and SFPTC), consistent with death of melanocytes, and disturbance of lipid metabolism in vitiligo lesions.

Some of the differentially expressed genes in the lesional skin were also differentially expressed in the nonlesional skin albeit to a lesser degree (**Supplemental Table 1**).

### SV and NSV Skin Lesions Are Indistinguishable at the Transcriptome Level

**Figure 1:**
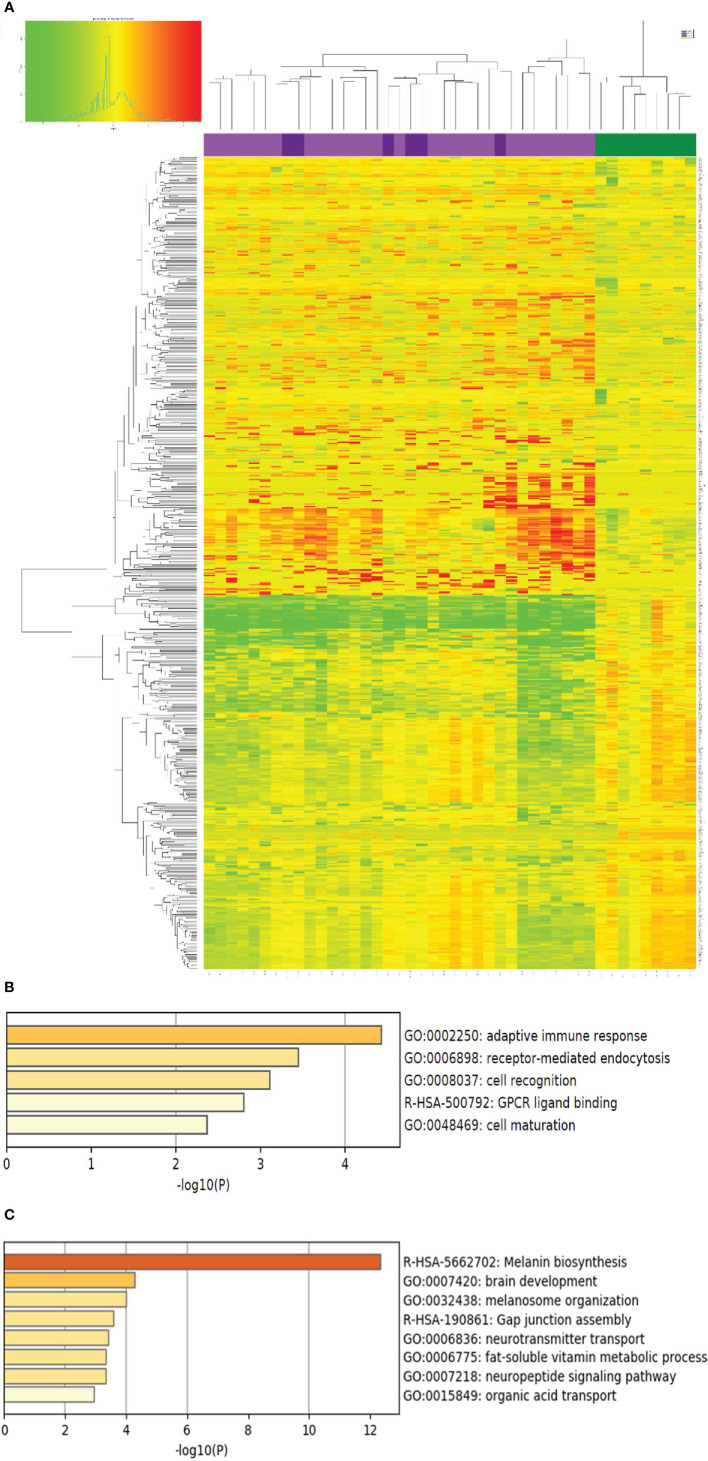
Genes with differential expression between vitiligo lesional skin and normal health skin. **(A)** The expression of the 723 genes with significant differential expression (> 2 fold change, p<0.05) compared with normal healthy skin (NHS, shown in green on the top panel, N=9), lesional skin of segmental vitiligo (shown in dark purple, N=6), and non-segmental vitiligo (bright purple, N=30) is presented as a heat map with non-supervised clustering analysis. Red and green color designate up or down regulation, respectively, of the gene in that particular sample compared to the average of healthy normal skin. The complete list of these genes are included in **Supplemental Table 1**. **(B, C)** Biological pathways represented by genes up and down regulated in LS over healthy normal skin, respectively. The p values were obtained by Metascape analysis.

As shown in **Figure 1**, the lesional skin of SV and NSV were not separable at the transcriptome level, suggesting that despite the stark contrast in the lesional distribution, SV and NSG are similar at the molecular level based on this small sample size comparison involving 6 SV patients and 30 NSV patients.

### Melanocyte-Signature Genes Show No Significant Association With Therapeutic Response or Vitiligo Duration

**Figure 2:**
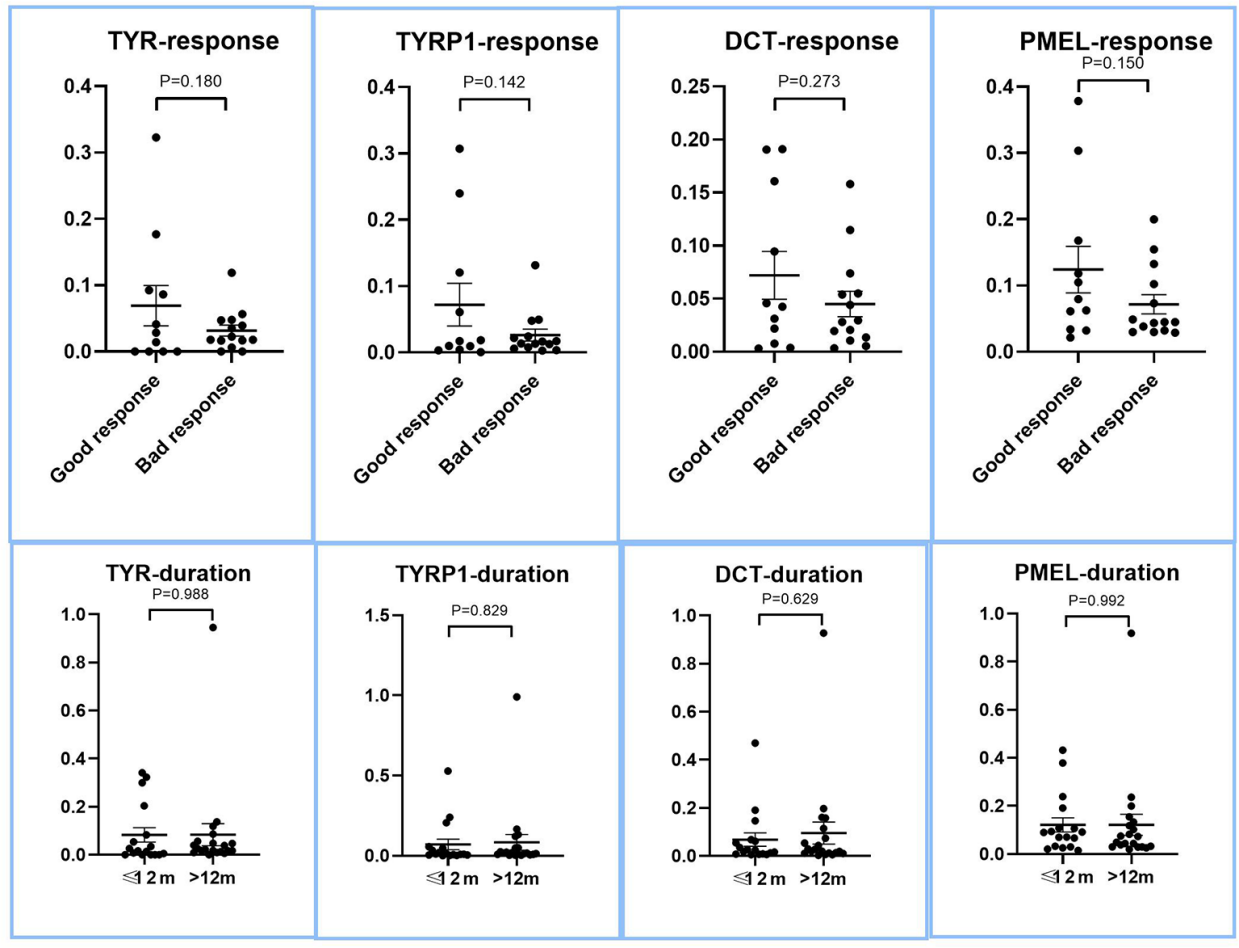
Expression of melanocyte marker genes in lesions with good and bad response to therapy, and between long and short duration lesions. The LS/NLS expression ratios of major melanocyte marker genes, DCT, TYR, TYRP1, and PMEL of good and bad response groups are presented as dot plots. There was no significant relationship between the expression levels of these genes and therapeutic response (p > 0.05).

Given that recent research showed that preservation of perifollicular pigmentation under dermatoscope (47), we evaluated if there were more residual melanocytic biomarkers in lesions of good therapeutic response or short duration. As shown in **Figure 2**, all of the melanocyte marker genes showed a trend of higher residual level of expression in good response and short duration lesions, the degree of higher expression did not reach the level of statistical significance (p>0.05, two tailed t test), suggesting the level of residual melanocytic marker genes cannot be reliably used to predict therapeutic response.

### Biomarkers of Vitiligo Therapeutic Response to NBUVB-Tacrolimus Combination Therapy

**Figure 3:**
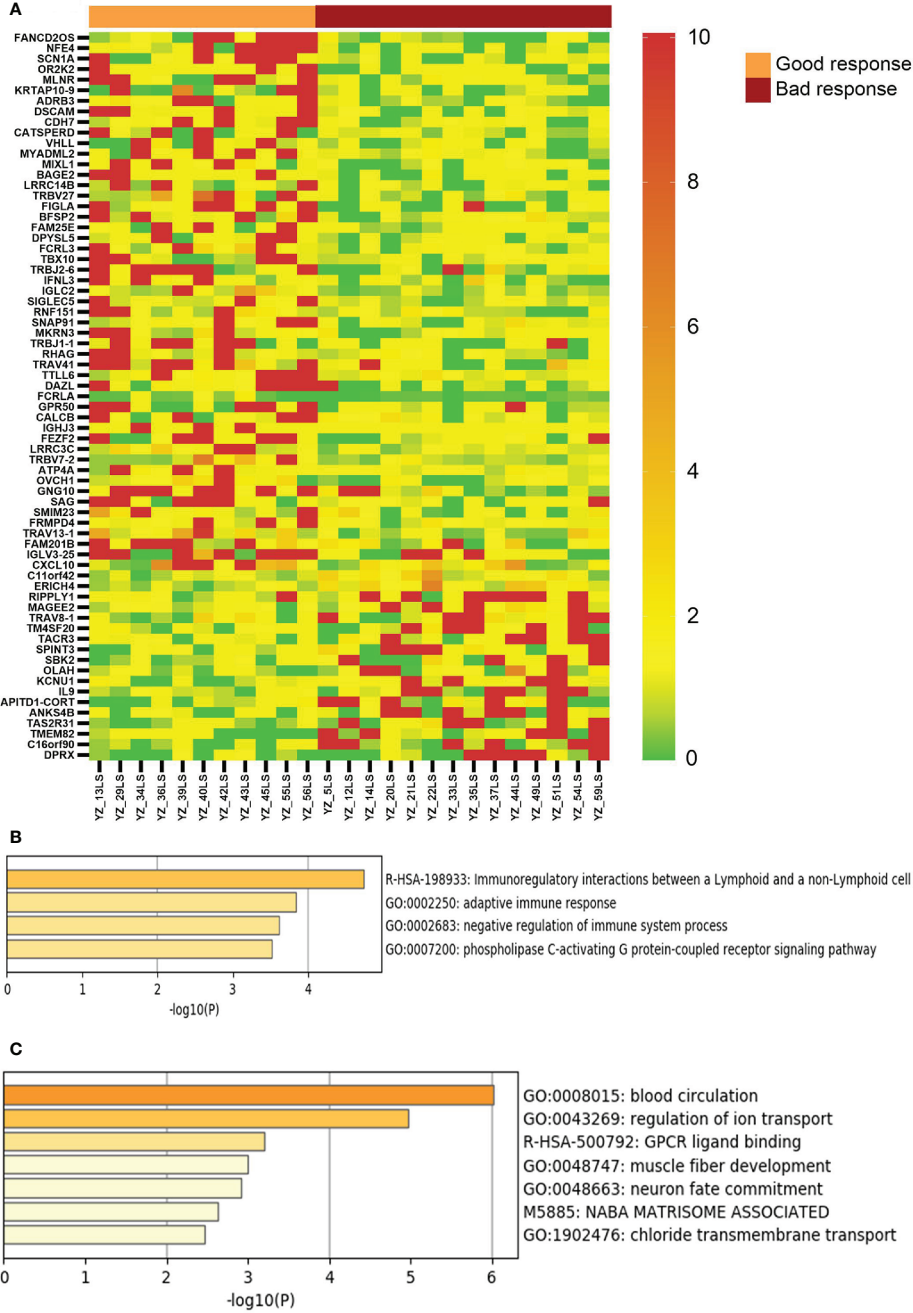
Genes with differential expression between vitiligo lesions with favorable response and lesions with non-favorable response to therapy. **(A)** LS/NLS ratios of 69 genes showing >2 fold difference between good response and bac response lesions (p < 0.05); **(B, C)** Pathways represented by markers of good and bad response, respectively.

We then compared the transcriptomes of the responsive and non-response vitiligo patients, and identified 68 genes with >2 fold differential expression (average level of each group, p<0.05, two tailed t test) between these two groups (**Figure 3**). Sixteen of the 50 up-regulated genes in good response lesions are involved in regulation of immune response (such as CXCL10, FCRL3, and T cell receptor genes). These 68 genes were able to completely separate the good response lesions from the bad response lesions, demonstrating the potential of them being used for therapeutic response prediction in the future.

### Biomarkers of Vitiligo Disease Duration

**Figure 4:**
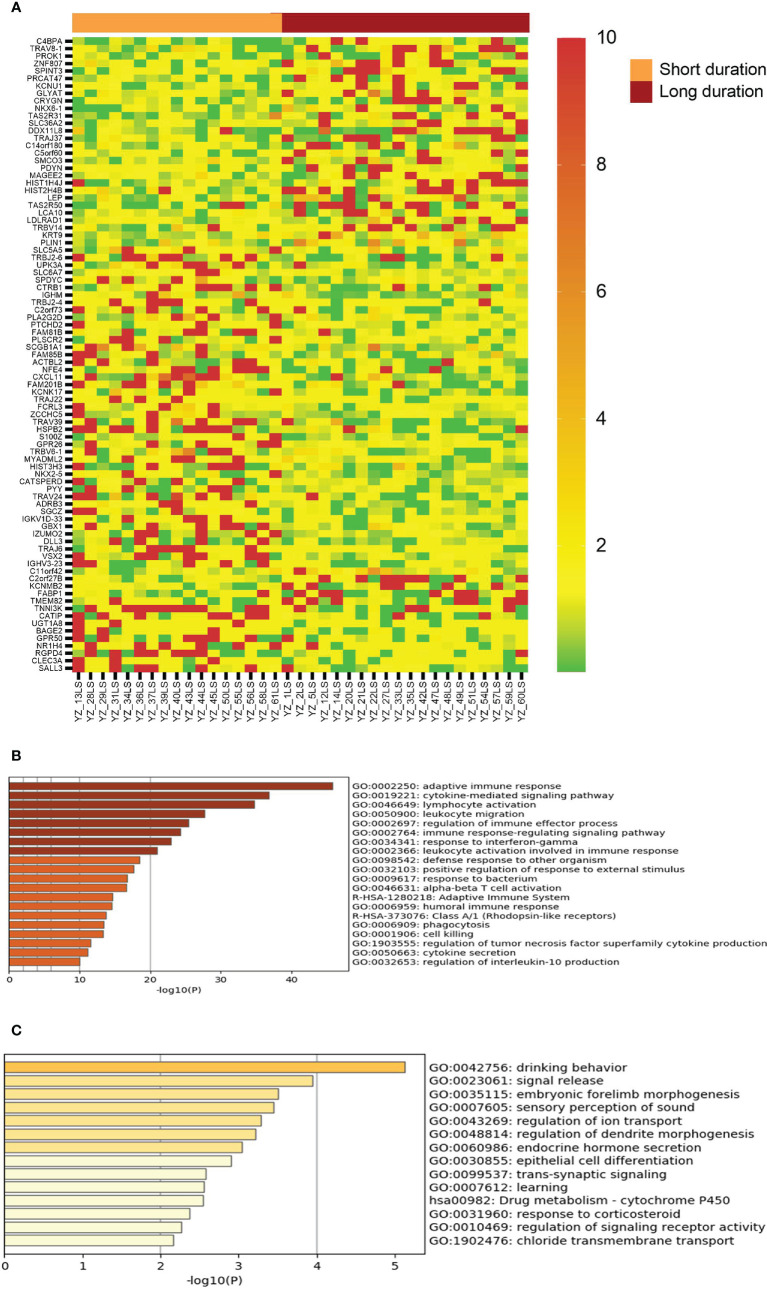
Genes with differential expression between lesions with short duration and lesions with long duration. **(A)** The LS/NLS ratios of 85 genes with >2 fold up or down regulated between lesions of <12 month duration and lesions >12 month duration. **(B, C)** Top biological genes enriched or decreased in short and long duration lesions, respectively.

Given that vitiligo lesions with shorter duration have better response to NBUVB-tacrolimus therapy, we examined the genes showing significant differential expression between duration less than 12 months and duration longer that 12 months. As can be seen in **Figure 4**, vitiligo lesions with shorter duration express significantly higher levels of genes in the regulation of adaptive and cellular immunity, including some of the genes associated with good therapeutic response such as CXCL10, FCRL3, and T cell receptor genes.

## Discussion

At present, several clinical parameters have been associated with more favorable response to vitiligo repigmentation therapies, including pediatric age, facial location, short disease duration, and absence of leukotrichia (poliosis) (48, 49). Since our study focused on patients with target lesions without leukotrichia that are mostly from torso and proximal extremities, our study was not designed to evaluate the impact of location on facial or acral sites, or leukotrichia on therapeutic responses. Only three of our patients were of pediatric age (two from the good response group and one from bad response group), therefore, our study was not designed to yield information on the impact of pediatric age on therapeutic response. We performed additional analyses excluding these three patients (data not shown), the results and the conclusions did not change.

Further, since we did not evaluate glabrous vitiligo lesions, this study could not generate information on the impact of hair follicles on therapeutic response. Our study did confirm that shorter duration is associated with better therapeutic response to NBUVB-tacrolimus combinations therapy. However, since we did not collect information on lesional stability art baseline, we could not comment on if the short lesional disease duration was correlated with disease activities such as lesional progression or regression.

The relationship between lesional skin pigmentation and the patient’s therapeutic response to vitiligo therapy has been unclear as the existing literature provided conflicting conclusions. On the one hand, vitiligo lesions with peri-follicular pigmentation under dermatoscope is associated with more favorable response (47). On the other, the degree of depigmentation in general did not show correlation to NBUVB therapy (50). We systematically evaluated the level of residual melanocyte-signature markers such as DCT, TYR, TYRP1, and MLANA in lesions of good and bad therapeutic response. Although there was a strong trend that good response lesions tend to have higher levels of melanocyte-signature gene expression, there was a wide range of variation, making the difference non-significant statistically. This suggests that melanocyte-signature genes are not robust markers of therapeutic response.

Our study attempted to take a novel approach to identify parameters associated with therapeutic response by performing transcriptional sequencing of vitiligo patients’ lesional and non-lesional skin, and identified a panel of 68 genes that could completely differentiate the vitiligo lesions with good response to therapy from those with bad response. It is of interest that these response biomarker genes include many with functions in immune activation, including CXCL10, which had been shown to play critical roles in the development of vitiligo (17). This discovery showed that there are still residual subclinical immune activity present in established skin lesions and that these lesions are more responsive to NBUVB and tacrolimus therapy, which are known to be immune suppressive in nature.

Previous studies showed that short disease duration is associated with better response to vitiligo therapy, which was confirmed in our study (**Table 2**). Further, our results demonstrated an inverse relationship between vitiligo lesional duration and the level of persistent immune response, including some of the immune biomarkers associated with good therapeutic response such as CXCL10, FCRL3, and T cell receptor genes.

Taken together, our study showed that in order to maximize the chances of therapeutic success for patients with vitiligo, one needs to initiate immune suppressive therapies, such as NBUVB and/or tacrolimus therapy as early as possible.

The current study has several limitations. First, the sample size is relatively small, with transcriptomic analysis involving 36 patients (only six with segmental vitiligo) and therapeutic response evaluable in 25 patients, limiting the statistical power of our study. It is possible that a larger sample sized study may uncover additional biomarkers that have not been detected in the current study. Another limitation is that we did not examine the biomarkers of the lesional borders, where inflammatory and immune activities are the strongest. Future studies will be needed to address this issue.

It should be pointed out that our study evaluated only a form of immune-suppressive therapy, NBUVB combined with tacrolimus, and did not directly address the therapeutic response prediction of surgical treatments of vitiligo lesions, which have been reported to have higher chances of success for lesions that are stable over time with no signs of recent lesional progression. We speculate that the favorable response biomarkers of immune suppressive therapy discovered in this study (high expression of immune response genes) would be biomarkers of non-favorable response to surgical therapies of vitiligo.

In addition, since this study was designed to evaluate skin microenvironment of well-established vitiligo lesions, the lesional and non-lesional skin biopsies were obtained at approximately 1 cm inside or outside the lesional borders, this selection criteria excluded vitiligo lesions smaller than 2 cm in sizes from being included in this study.

Further, our study was focused on the assessment of target vitiligo lesions’ response to therapy, we could not answer the question if biomarkers obtained by sampling one lesion would have predictive power for the un-sampled lesions of the vitiligo patients, which often have multiple lesions. Further studies are needed to address this question.

Our study involves obtaining full-thickness biopsies of the skin lesions, which makes it impractical for routine testing in the clinic. However, this shortcoming could be easily overcome in the future by modification of the sampling technique, capitalizing on the technical advance of using non-invasive techniques such as tape-stripping to quantify biomarkers of the skin (51–53).

Finally, this study also uncovered previously unknown gene expression changes in the skin of patients with vitiligo, including dysregulation of genes involved in lipid metabolism (such as SFPC and PCSK2). These indicate that the pathophysiology of vitiligo is more complex than what have been reported at present, such as loss of melanocytes. The functional and clinical significance remains to be further elucidated in the future.

In summary, this study showed that short disease duration is associated with better therapeutic response, and that higher expression of genes regulating innate and adaptive immune response are factors predictive of more favorable response to vitiligo therapies that are immune suppressive in nature. Therefore, early therapeutic initiation and suppressing persistent immune activities of vitiligo skin lesions are required to restore melanocytes in vitiligo.

## Data Availability Statement

The raw data supporting the conclusions of this article will be made available by the authors, without undue reservation.

## Ethics Statement

The studies involving human participants were reviewed and approved by Clinical Ethics Board of University of British Columbia. Written informed consent to participate in this study was provided by the participants’ legal guardian/next of kin. Written informed consent was obtained from the individual(s), and minor(s)’ legal guardian/next of kin, for the publication of any potentially identifiable images or data included in this article.

## Author Contributions

QY: Statistical analyses, statistical mapping, and drafting of the manuscript. GZ and JZ: Bioinformatics and statistical analyses. MS and GL: Biopsy collecting and RNA extracting. HL: Conceptual development of the project and therapeutic response data. PZ and JX: Bioinformatics and statistical analyses and critical revision of the manuscript. YW: Statistical analyses. XZ: RNA sequencing and funding. YZ: Patients enrolling, demographical information and clinical parameters, development of the project and therapeutic response data, drafting and revision of the manuscript, and funding. All authors contributed to the article and approved the submitted version.

## Acknowledgments

This study was funded in part by grants from Canadian Institutes of Health Research, Canadian Dermatology Foundation, National Natural Sciences Foundation of China, and Canadian Melanoma Foundation.

## Conflict of Interest

The authors declare that the research was conducted in the absence of any commercial or financial relationships that could be construed as a potential conflict of interest.
